# Lithic Landscapes: Early Human Impact from Stone Tool Production on the Central Saharan Environment

**DOI:** 10.1371/journal.pone.0116482

**Published:** 2015-03-11

**Authors:** Robert A. Foley, Marta Mirazón Lahr

**Affiliations:** Leverhulme Centre for Human Evolutionary Studies, Department of Archaeology and Anthropology, University of Cambridge, Cambridge, United Kingdom; Université de Poitiers, FRANCE

## Abstract

Humans have had a major impact on the environment. This has been particularly intense in the last millennium but has been noticeable since the development of food production and the associated higher population densities in the last 10,000 years. The use of fire and over-exploitation of large mammals has also been recognized as having an effect on the world’s ecology, going back perhaps 100,000 years or more. Here we report on an earlier anthropogenic environmental change. The use of stone tools, which dates back over 2.5 million years, and the subsequent evolution of a technologically-dependent lineage required the exploitation of very large quantities of rock. However, measures of the impact of hominin stone exploitation are rare and inherently difficult. The Messak Settafet, a sandstone massif in the Central Sahara (Libya), is littered with Pleistocene stone tools on an unprecedented scale and is, in effect, a man-made landscape. Surveys showed that parts of the Messak Settafet have as much as 75 lithics per square metre and that this fractured debris is a dominant element of the environment. The type of stone tools—Acheulean and Middle Stone Age—indicates that extensive stone tool manufacture occurred over the last half million years or more. The lithic-strewn pavement created by this ancient stone tool manufacture possibly represents the earliest human environmental impact at a landscape scale and is an example of anthropogenic change. The nature of the lithics and inferred age may suggest that hominins other than modern humans were capable of unintentionally modifying their environment. The scale of debris also indicates the significance of stone as a critical resource for hominins and so provides insights into a novel evolutionary ecology.

## Introduction

Humans have had and continue to have an enormous impact on the environment [1], to the extent that they are now influencing the climate itself. Major anthropogenic effects on the environment came primarily with agriculture and the larger populations it supported [2], leading to the ‘early Anthropocene hypothesis’ [3,4], but earlier impacts also occurred, such as the use and control of fire [5,6] and over-exploitation of large mammals leading to their extinction [7]. While the timing and extent of these have been contested [8], the gradual increase in human alteration of the Earth’s environment is indisputable [1,9,10].

However, extensive fire use and over-hunting were relative late-comers in the repertoire of human behavioural and ecological changes. One of the earliest and most significant was stone tool-making. When hominins first adopted stone tools as a significant element of their behaviour, the first modifications of the landscape took place through quarrying, the moving and breaking of boulders, and reducing cores to their final products. Here we elaborate on the nature of this first level of human impact on the environment and use a vast landscape in the Central Sahara (Fazzan, Libya), where the surface is extensively made up of man-made lithics, to explore the extent to which this may have occurred. We show that, for substantial phases of the Pleistocene, part of this landscape was used and fundamentally modified by hominin activities associated with stone tool production. The particular visibility of lithics in denuded landscapes, such as the Central Sahara, allows us to assess the extent of the human impact in the area. However, it also throws light on the long-term ecological effects of stone-tool making by hominins, one of the earliest forms of anthropogenic environmental change.

The fracturing of stone to make tools is a key element of human evolution, and the archaeology of lithics has provided the richest source of information about the remote hominin past. The first incontrovertible evidence for stone tool production is at 2.4 million years (Ma) from Gona in Ethiopia [11], although there are indirect suggestions of stone tool use before 3.0 Ma [12]. These very early instances of intentionally made artefacts are rare; however, after 1.8 Ma, the frequency of sites, density of lithics on sites and typological diversity all increase [13]. Different lines of evidence, from site formation, to the association of lithics with animal carcasses, to the first trans-continental hominin dispersals, indicate that, by this stage, some hominin species were dependent upon stone tools for survival (as opposed to ephemeral, opportunistic or local population-specific use, as in chimpanzees [7,14][8,15]. By 1.76 Ma, hominins were making not just Mode 1 (Oldowan), but also Mode 2 (Acheulean technologies) [16] stone tools, which become a prominent part of the African and western Eurasian landscape after about 0.8 Ma [17]. Further major changes in technology, in the form of Mode 3 industries (Middle Stone Age, prepared core, Levallois), took place possibly as early as 500 thousand years ago (Ka) [18,19] and certainly by 280 Ka [20], and are widespread in Africa and beyond [21]. The technologies of the Upper Pleistocene become increasingly variable and widespread. There are thus nearly two million years of significant and increasing lithic exploitation and discard across many parts of Africa.

The increasing importance of stone tool use (and presumably similar use of other materials) can be explored in terms of questions about the cognition of the makers, the way in which lithic artefacts can enhance subsistence strategies, and also affect how hominins used the landscape. Important to all of these is the nature of the raw materials available. The size and quality of the raw material can affect how flexibly a hominin could reduce the core to a desired goal, how effectively it would work as a means of acquiring or processing resources, and its distribution can limit or promote use of particular areas.

These research studies have tended to examine the way in which access to good quality stone influences human behaviour and adaptation [22,23]. There are, however, important questions to be addressed in the other direction—how did hominin use of and increasing dependence upon stone as part of their adaptive strategies have an impact on the environment? It has been demonstrated that life in general has an impact on the lithosphere and topography[24]; a further question is whether prehistoric people, through their use of stone, also left a tell-tale signature. Fewer studies have contributed answers to this question.

Data from extensive surface surveys can be used to provide an indication of the scale of discarded lithic debris across various landscapes (Table A in S1 File). For example, over a variable (but primarily mid-Holocene) environment of 600 square kilometres (km^2^) in Amboseli (Kenya), an average of 19,000 lithics per km^2^ was recorded [25]. Hardaker reports on 16 sampled areas in the Zebra River Valley in Western Namibia, with an average density of 2.3 lithics per m^2^ (excluding a significant outlier), which is the equivalent of over two million per km^2^ [26]. Isaac, in a survey focused on specific artefact-bearing Plio-Pleistocene sediments in Koobi Fora, found a baseline density of 40,000 lithics per km^2^ [27]. The high Nubian Desert yielded densities of between 1–12 x 10^6^ per km^2^ [28]. In visual terms, this means that one can expect to encounter a prehistoric lithic artefact in some areas of Africa every m^2^, and in others every 50 m^2^.

These data come from areas where archaeological visibility is high, and where landscapes are eroded, and would not reflect observable densities more widely. To work around the issue of landscape taphonomy, the problem can be approached from the other end—the behaviour of prehistoric hominins. Although there is only limited ethnographic evidence of rates of lithic manufacture among stone using peoples, we can make some broad estimates of rates of extraction and production (see Methods).

Confining such a model to Africa (approximately 30 million km^2^), as the continent with the longest record of stone tool use [11], we can assume, conservatively, a Pleistocene density of one person per 100 km^2^, and less conservatively of 1 person per 10 km^2^ across the continent for the period of stone tool using human evolution [29]; we can propose a minimum production rate of 10 tools per year, to a maximum of 100. To this we can add the additional debris produced per flake (débitage); even an Oldowan (Mode 1) reduction may comprise nearly 100 flaking events [30], and experimental data on Acheulean (Mode 2) and Levallois (Mode 3) yield an average of 63.9 flakes (larger than 2 cm) per reduction sequence [31]. For the purposes of the modelling here, a minimum of 10 waste flakes per reduction event, as a very conservative estimate, to a maximum of 100, is used.

This yields a very unrealistic minimum (allowing for no additional débitage) of 3 million lithics produced in Africa per year, or 3 x 10^12^ over a one million year span (used here as a conservative figure, although for some areas of Africa this timespan may be considerably longer); a maximum, based on the higher densities, production rates, and débitage levels, would yield 3 x 10^9^ per year, or 3 x 10^17^ across a million years. Spread evenly across Africa, these minimum and maximum models would yield a density of between 10^4^ and 10^9^, per km^2^. A very conservative—and unrealistic—estimate would be as low as an average of 10,000 artefacts per km^2^_._ If we exclude the upper and lower extremes, the models suggest an average density of between 10^6^ and 10^7^ per km^2^ (i.e., between one million and ten million artefacts per km^2^, which would be close to the highest observed density of the Nubian Desert (12 million per km^2^). Given the complex and variable ecology across Africa today and in the past, and an uneven hominin distribution, a more realistic estimate would be to consider the prehistoric distribution of stone tools over half the continent’s surface, yielding an average density of between 0.5 million and 5 million artefacts per km^2^.

We can turn these figures into volumetric estimates. Let us assume that the average volume of a piece of débitage is 7 cubic centimetres (5 x 3 x 1 cm). Taking the maximum figures, this yields 2.1 x 10^20^ cubic centimetres, or 2.1 x 10^14^ cubic metres of rock. This is the equivalent of 84 million Great Pyramids of Giza (which is 2.5 x 10^6^ cubic metres [32]), or 42 million taking into account the uneven hominin occupation suggested above. To extend the comparison further, it would be the equivalent of finding between 1.3 and 2.7 Great Pyramids per square kilometre throughout Africa.

We do not have sufficient archaeological information to refine these estimates in relation to different time periods, although one can hypothesise that the extent of stone tool use was limited—as was hominin distribution and density—in the period prior to 1 Ma; that the very high density sites known for the Acheulean after 1.0 ma would indicate considerably heavy use of stone, especially in relation to the large size of the artefacts; and that use of Mode 3 technology is also likely to have represented a zenith of number and volume of material. With the industries of the Upper Pleistocene, it is likely that higher population densities, greater technological complexity and reduction in lithic size would have increased the number of artefacts, if not the volume of raw material used. In this context, we can expect, if there is any environmental impact, it will be in place before the end of the Middle Pleistocene.

These figures may still be highly conservative, and underestimate the sheer quantity of waste product involved in stone tool manufacture. Where good stone is very common, usage may be higher. Primary preparation of large boulders to create the cores from which artefacts are made can increase the volume of stone extracted massively, and give rise to the expectation that the potential for early humans to modify the landscape through their dependence upon stone is very considerable.

Given this scale of activity and exploitation, the extraction of raw materials for lithic production has the potential to have an impact on the environment. Palaeolithic quarries have been identified [33–36], some on a very significant scale [37,38]. These, however, are local outcrops or beds, rather than complete landscapes. The Messak, a silicified sandstone massif in the Libyan Central Sahara, provides an exposed, undisturbed prehistoric landscape, where the opportunity to explore such an impact at a regional level is possible.

**Fig 1:**
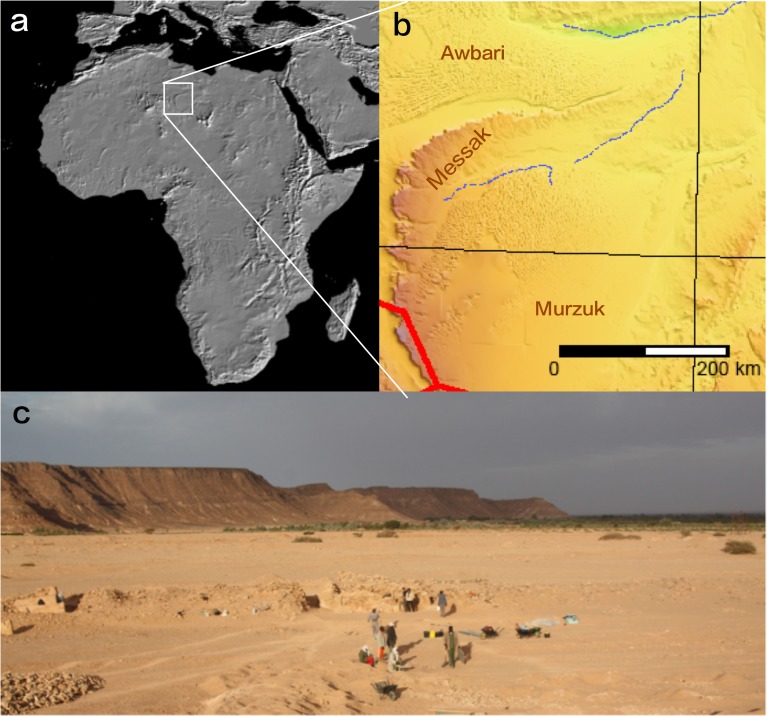
Location of Messak. a) The Messak is located in the Libyan Central Sahara (Fazzan); b) the Messak is a massif of sandstone that lies between two sand seas, the Awbari to the north, and Murzuq, to the south (source: Google Earth); c) view of the Messak from the south, close to the town of Jarma with excavations of the Garamantes Royal Cemetery by DJ Mattingly and team in the foreground. Sources: Images: Elevation map of Africa from Nasa (http://photojournal.jpl.nasa.gov/catalog/PIA04964), detailed map of Fazzan adapted from Wikimedia Commons (http://commons.wikimedia.org/wiki/File:Libya_Topography.png); photo of Messak by M Mirazon Lahr.

As part of the Desert Migrations Project, a program of research into the prehistory and proto-history of the Central Sahara (Fazzan, Libya) [39–42], we carried out intensive surveys of the Messak escarpment (Fig. 1), on the surface of which lithics were littered on such a scale that it can be described as a man-made landscape (see Figs. B and C in S1 File).

## Results

**Fig 2:**
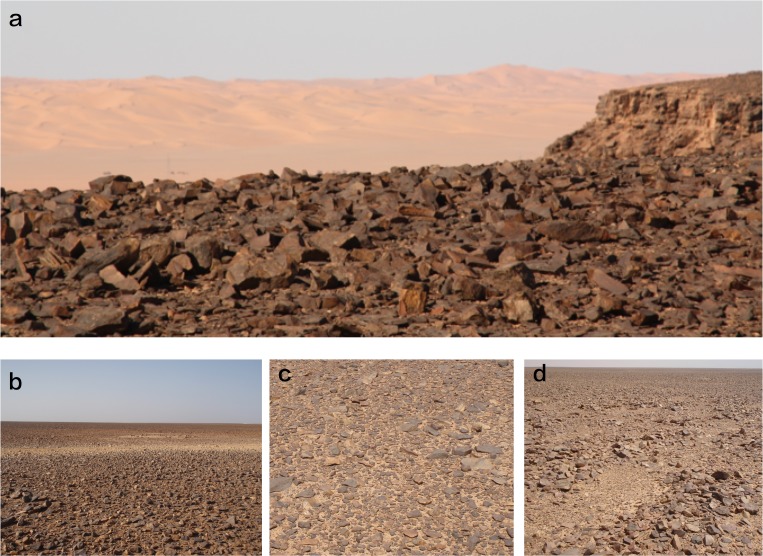
The Messak landscape. The Messak is almost entirely without soil or superficial sediments, other than recent Aeolian deposits from the adjacent sand seas (a). The surface is made up of broken rocks (b, c, d), many of which have been humanly modified. In some cases (c), the removal of rocks has led to small depressions that can become small puddles or ponds. Sources: photos by authors.

The Messak is a Cretaceous massif oriented N-S on its western side, and ENE-WSW in its northern part. It is located between 23.4o and 26.5o N, and 11.3o to 13.3o E, and lies between the Awbari and Murzuk Sand Seas (Fig. 1). The massif is shaped by a sharp escarpment of up to 300 m to the north and west, sloping gently to the south and east. It is divided into two main units—the Messak Settafet and the Messak Mellet, with a total length of circa 350 km and average width of 60 km, thus creating a 15,000 km^2^ formation of sandstones and shales [43,44]. The surface is dissected and incised, with virtually no remaining recent sedimentation. It is probable that major erosion has occurred in the recent past, stripping the Messak to a rock and gravel-strewn reg [44]. The sandstones of the Messak Settafet, which are ubiquitous, are highly silicified and provide a high quality raw material for making stone tools. The most striking aspect of the landscape of the Messak is that stone tools occur ubiquitously, constitute a significant portion of the surface cover, and in places the environment is completely dominated by this prehistoric activity (Fig. 2, Figs. B and C in S1 File). We know that there is abundant evidence for the presence of hominins in the Sahara from the Lower Pleistocene onwards. This relates both to the area as a route for dispersals beyond sub-Saharan Africa, and also a place that may have been highly habitable during the wetter phases of the last million years [45–48]. It is also the case that the Sahara represents an enormous area, with contrasting biogeographical links[48], and the potential for considerable structure within[49].

**Fig 3:**
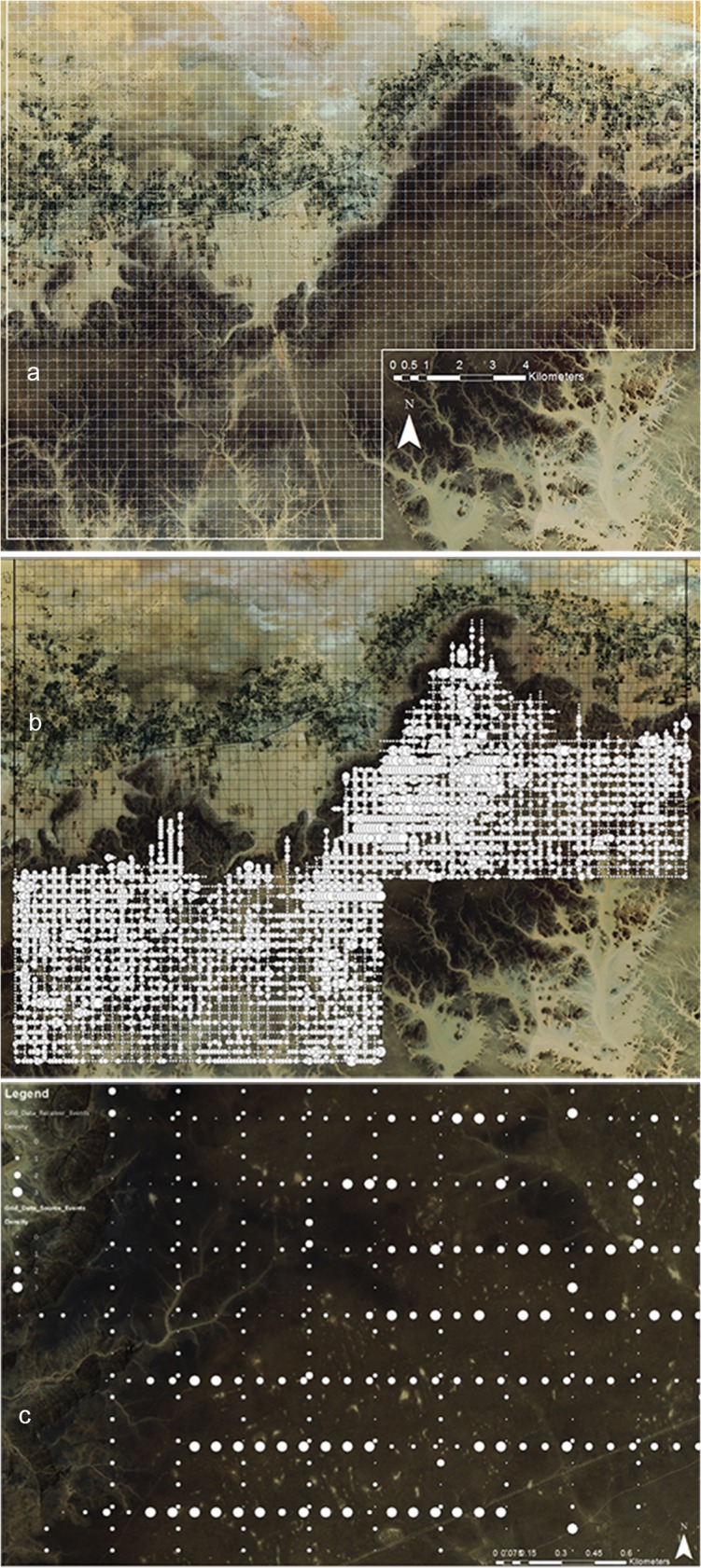
Frequency and distribution of lithics on the Messak as recorded in the OXY survey. a) Satellite image of the Messak, showing the seismic grid lines laid for the oil exploration. Each of these lines was walked by archaeologists from the Libyan Department of Antiquities, in both directions. Major archaeological localities were recorded on and between the lines, and the presence of lithics and rough density recorded in 2 x 2 m box every 100 m along the lines. b) Artefact density across the 2 x 2 boxes from the survey; white circles indicate presences of lithics, with the size reflecting low, medium and high density. c) Detail of a small 3 x 2 km area from within the survey area on the plateau’s surface, showing the presence and density of lithics. Sources: MESSAK SURVEY BACKGROUND MAP: satellite image donated by Occidental Plc. (WGS 1984 UTM Zone 33N, Extent: 2960788.37_291786.562_326263.362_2913482.77)

The very high density of stone tools can be measured at several levels (Fig. 3). Across the Messak as a whole, stone tools are extremely common, and any survey will encounter multiple scatters and isolated artefacts [50] [39–41,43,44,51], suggesting widespread use of the local sandstone as a source of raw material. At a more local level, stone tools are spread densely and extensively. In a very intensive survey carried out as part of a heritage assessment prior to oil exploration over >400 km^2^ in 2008 [52,53], 2 x 2 m plots were sampled every 100 m on transects running both east-west and north-south on ∼140 km^2^ of the Messak, with 300 m between transects (Fig. 3 and see Methods for details). The result was a lattice of plots every 100 x 100 m along the lines, with a density assessment at 8232 points, of which 6090 points were on the plateau surface of the Messak (i.e., excluding wadis). Of these 6090 sample points, lithics were identified in more than 60% including 17.6% with moderate to high density (Fig. 3). These data indicate a minimum density of quarter of a million lithics per km^2^.

In the 2008 Survey [52], for logistical reasons, the archaeologists walking the survey lines only recorded the presence of visible and diagnostic artefacts, rather than total débitage. Consequently, these data show a distribution of surface lithics biased towards large and easily recognizable artefacts, and so represent minimal or relative densities only, not a true density. To obtain a measure of the real extent to which the stones on the surface of the Messak were humanly modified, we carried out a further survey in 2011. Fifty quadrat plots of 1 m^2^ were randomly selected on the plateau surface, distributed across five areas (Fig. A in S1 File). Within each quadrat we recorded the number of stones > 15 mm in length, and identified the number of these which showed evidence of having been humanly modified. The artefacts were all made on local sandstones, and varied from very diagnostic cores and tools, mostly indicating Mode 2 or Mode 3 industries, to undiagnostic flakes and débitage. The material was virtually all macro- rather than microlithic (Figs. B and C in S1 File).

The results are striking (Table 1 and Table B in S1 File). All of the quadrats show a high density of stone material. The average number across all the fifty samples is 282 (SD = 118, range = 64–593). A very large proportion of these show signs of having been modified by hominin activity. The average number of artefacts across all the samples is 75.22 per m^2^ (SD = 24.1, range = 37–168). The mean proportion of lithics to unmodified stones in the quadrats is 31.3%; the range varies from 7% to 84%. There is no statistically significant relationship between the number of lithics and the number of unmodified stones in the quadrats, although it is probable that this would be strongly influenced by size—as the number of small pebbles increases, the proportion of lithics goes down.

**Table 1:** Numbers of stones and lithics per sample quadrat (1 m^2^ each).

Area	Sample number	Mean number of stones > 20mm	SD	Mean number of lithics	SD	Min	Max
A1	10	188	52.5	70.8	17.8	37	102
A2	10	320	82.9	81.9	20.2	46	112
A3	10	428	87.4	84.9	36.9	44	168
A4	10	299	74.3	68.1	19.1	37	94
A5	10	178	81.1	70.4	21.3	53	125
Total	50	282	119.0	75.2	24.1	37	168

Ten one metre quadrats were sampled in five areas.

**Fig 4:**
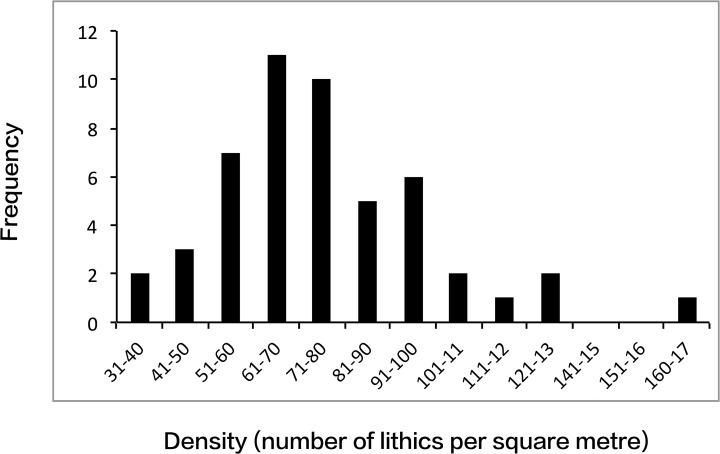
Frequency distribution of artefact densities in the 50 1 x 1 m sample squares.

There does not appear to be any strong geographical patterning between the five different areas sampled (minimum area average is 68.1 lithics, maximum is 84.9). Although the area sampled was confined to one part of the Messak, previous extensive surveys across the escarpment as part of the Desert Migrations Project and by others [39–41,43,44,50,51] indicate that this is typical for the region as a whole. Although the variation between sample squares is high (37–168), it is normally distributed, and the high average number of artefacts recorded is not the result of a few high density outliers (Fig. 4).

## Discussion

The primary conclusion is that the current surface of the Messak is, in effect, man-made. The mostly Mode 2 and Mode 3 technological status of the diagnostic lithics (SI.4) would place this in at least the Middle Pleistocene (0.9 to 0.125 Ma), and perhaps, given the older dates for the Acheulean in Africa [54], even older. The absence of significant evidence for Upper Palaeolithic or Later Stone Age occupation in the area would support the idea that the anthropogenic landscape had been formed, through palimpsest build-up, probably by at least 100,000 years BP, and certainly by 50,000 years BP. This chronology would make the Messak one of the oldest humanly modified landscapes on a geographical scale greater than that of individual quarries.

The age and scale of the environmental impact throws light on the nature of hominin ecology. First, the scale of activity is such that it is clear how important stone tool activities were for hominins, and it shows that African Pleistocene *Homo* was a strongly technologically dependent genus, and its distribution would have been as limited by the availability of stone as of any other resource. Second, landscapes such as these must have been magnets for hominin populations, either for ‘stone foraging trips’ or for residential occupation. Large parts of Africa provided these resources—the East African Rift, the Saharan outcrops and plateaus, the silcrete regions of southern Africa—and this may have been a factor in patterns of hominin dispersals and biogeography. The scale of activity also raises the question of contrasts between Africa and many parts of Asia, where rich lithic landscapes are comparatively rare [15]. Is this a question of differences in degrees of technological dependence among hominin, or the limits of raw material availability and the use of alternative resources? And third, at locations such as the Messak, the superabundance of stone is likely to have made the extraction techniques flexible and pragmatic, and there is little evidence of careful husbanding of resources; while diversity of reduction techniques exists, it still remains within the normal range of variation for Early and Middle Stone Age technologies in other parts of Africa.

Two further observations can be made in relation to extraction processes—one that they are variable, the second that they can affect local ecology. On the Messak, lithic extraction took place in a number of ways, some showing simple opportunism, others more systematic and intensive behaviours. At the simple level, large boulders may have been the subject of simple, opportunistic removal of large flakes. At the more sophisticated level, a technique similar to feather and wedge may have been used. Large worked boulders can be seen with numerous small stone wedge-shaped flakes, and incomplete breaks have a quantity of surrounding small fragmented debris that can be interpreted as the wedges used for breaking up the largest boulders. In some cases, existing cracks in a boulder are enlarged by placing small stone wedges and using these to expand and complete the fissure. Other techniques may well have been used, and in addition, special raw materials, such as fossil wood, was selected and exploited [39].

The second observation is that the exploitation of the stone may have influenced the local ecology. One method used was to remove large pieces from the surface, and so create small quarrying pits. These occur in clusters, and can be 1–2 m in diameter, and 20–50 cm in depth. They may be spaced relatively close to each other (∼2 m), or more widely dispersed, but are not continuous across the landscape. In some small patches, more than 90 such quarrying pits were observed over an area of about 10,000 m^2^. More normally they would occur in small groups, their location determined by a patch of suitable raw material, but isolated pits were observed as well. These localities bear the scars of extraction, so that it is not just the addition of a humanly created debris, but an actual change in the landscape itself (Fig. D in S1 File). Given the enormity of the availability of the raw material, it is unlikely that the Messak environment was significantly ‘damaged’ in this process, but it may have had ecological consequences. Many of the areas of depression, whether pits or larger extraction surfaces, became small pockets where moisture was retained, and surface water exists today after rains. As such the extraction of the stone materials may not only have altered the environment negatively, it may also have enhanced the productivity of the area, and formed small pools that would have attracted game long after the original extraction event took place. It is noticeable that in many of these we find trapping stones [52]—large stones which were used for traps or ties for game and/or cattle, during the Holocene [55].

The Messak survey, in combination with other large scale surface studies, reveals the level of activity on the landscape by people rightly called ‘stone age’. The observed densities in the sample quadrats can be extrapolated for comparison with the observations and models based on previous work. The average density of 75 flakes per m^2^ extrapolates to a density of 7.5 x 10^7^ per km^2^—six times greater than the maximum density observed during the Nubian Desert study [28]. The figure also lies within the orders of magnitude ranges of the theoretical models developed in this paper (10^7^, compared to the maximum modelled range of 10^4^–10^9^, and the more conservative range of 10^6^ to 10^7^). This congruence between model and observation may indicate that when a fully exposed landscape is observed, we can see the impact; in many areas this has either been rendered invisible or destroyed.

Finally, we can consider briefly the implications for understanding hominin behaviour in prehistory. Perhaps the most important of these is whether super-abundant raw material patches such as the Messak were magnets to draw communities into an area. If, as seems likely, the success of a community depended to any significant extent on its use of stone tool technology, then there would be enormous advantage in identifying, knowing and remembering such localities (they would become significant landmarks), but also advantages in controlling access to them. In this respect, the value of the resource, ubiquitous as it is locally, increases in relation to the paucity of availability in the region (the sand seas). In this sense, the Sahara, in lithic terms is a very patchy environment, with the implications for behavioural models.

Another way of looking that this is in terms of tethering. All organisms are tethered to areas that hold essential resources—water being an obvious one in relation to hominins as well as many other species. If hominin survival became dependent upon lithic-based technology, then hominins would, in effect become tethered to areas where such raw materials are available. Differences in technological patterns across Africa and Eurasia during the Pleistocene show that is was unlikely to have been a universal pattern, but it may be the case that rich lithic areas such as the Messak would have promoted tethering, and made other adaptations and responses to environmental change subservient to that need.

The Messak is a striking element of the landscape, and must have been so even in a greener Sahara. We know very little about how early hominins would have perceived such an environment, even less responded to environmental change. It may be that hominins recognised the link between the creation of depressions that retained water, and so attracted game, but there is no direct or indirect evidence for intentional change, and one must parsimoniously infer that any enhanced environmental resources that occurred were, at best, unintentional niche construction. The consequences may have been real, nonetheless.

These data provide insights into what must have been an extraordinary level of activity by hominins. In a context of almost ubiquitous availability of raw material the environment was exploited very intensively. The impact is such that the surface of the Messak is effectively man-made, over tens of square kilometres, if not hundreds. This would represent humanly induced environment change of a large geographical scale, probably during the Middle Pleistocene—certainly among populations of humans and other hominins living entirely by hunting and gathering.

The Messak may be an unusual—but by no means unique—environment in having such a rich resource of lithic raw material and such high archaeological visibility. There is the possibility—given the ubiquity of material—that the sub-tropical belt stretching from the Sahara to Arabia offered unique opportunities and demands in relation to lithic raw materials, but it is more likely that what is unusual is the high level of ancient landscape visibility. Regardless, the richness of the archaeological record there is testimony to the extent of human technological dependence by, at least, the Middle Pleistocene, and so the extent to which the distribution of stone raw materials was a limiting factor in hominin adaptation. The lithic landscape also indicates how prior to agriculture, and independent of foraging behaviour, humans were capable of modifying the environments in which they lived.

### Methods

#### Field methods

The data used here come from two field seasons, and two different field procedures.

Density sample squares.

In 2011, five small areas on the Messak were selected, representing different local landforms—mesa flat top, edge of down-cutting, wadi, gravelly surface. In each of these areas, 10 one metre square quadrats were randomly sampled. Within each quadrat, all stones larger than 2 cm (longest dimension) were counted. All stones showing traits of diagnostic human modification stones were counted. Data in Table 1 and Table B in S1 File are in numbers of artefacts per square metre.

Intensive Block 131 survey.

In 2008, as part of an environmental impact assessment project carried out in relation to oil exploration by Occidental, an intensive archaeological survey was carried out. Across an area of >400 km^2^, lines in N-S and E-W directions, spaced 300 m apart, were walked by archaeologists from the Libyan Department of Antiquities, who identified > 3000 archaeological sites of varying extent. The Libyan archaeologists who took part on the survey were trained on the identification of Palaeolithic artefacts at the outset of the work. One hundred and forty four km^2^ of the surveyed area was on the Messak, and that is the area used here to calculate densities. Using a wider scanning method both along and between the lines, areas on the Messak with high densities of lithics and/or other archaeological remains were recorded, and photographs of the most diagnostic pieces taken, allowing the spots to be allocated to particular technological phases. This process identified ∼ 1230 sites and significant lithic scatters. In a more systematic survey, lithics and other remains were recorded in 2 x 2 m square sample boxes every 100 m along the seismic lines in both directions. Presence of lithics, with a focus on large and/or diagnostic pieces, was recorded (absence, low density, moderate density, high density).

#### Estimates of stone tool production

In the estimates of how many lithics were produced by ancient human populations in Africa a number of approximate calculations were made to set the bounds of likelihood. The parameters used are listed below (abbreviations in brackets).

1. Landmass of Africa—30,221,000 km^2^ (30 million used for convenience). (*A*)
2. Population density—based on extant hunter-gatherers, with 1 person per 100 km used as a minimum, and 1 person per 10 km as a maximum. (*D*) [29]
3. Length of time: although stone tool manufacture is known to extend back at least 2.5 Ma, its frequency and distribution in Africa in the early phases was probably low. For the sake of simple modeling used here, a duration of one million years of ubiquitous stone tool making is used, and more realistic models would probably increase this number, although the rate would change considerably across time and this should be factored into more refined models. (*T*)
4. Number of flakes used. This is a simple estimate of the number of tools required by one person in the course of a year. This is estimated a minimum of 10, which might be apprriate appropriate for the earliest tool-makers, and a maximum of 100, which may be conservative for later parts of prehistory. (*F*)
5. Débitage. To produce a flake requires reduction of a core, which can involve a large number of additional flakes and débitage being struck off. For the model used here a minimum of 10 pieces of débitage per tool, and maximum of 100 was estimated (*G*) [30] [31]
6. Volume. Stone tools can vary enormously in size, so that this parameter that should be modelled in relation to a distribution of sizes, which would require experimental data. A lithic can be a small chip struck off a core, or a large rock off which one flake has been removed. For simplicity purposes, a simple standard flake is used, measuring 50 x 30 x 10 mm. (*V*)

All estimates used in the model are conservative, so as not to overestimate the models and exaggerate the effects discussed in the results here. The following calculations were made, using the maximum or minimum values for any of the parameters, where *N*_*y*_ is the number of lithics produced in Africa per year, N_t_ *N*_*t*_ the number of lithics produced over the entire period, *N*_*v*_ is the volume (in cubic metres) of lithics in total, and *N*_*D*_ is total expected density in Africa per square kilometre

$$N_{y}=\left(AD\right)\left(FG\right)$$

$$N_{t}=\left(AD\right)\left(FG\right)T$$

$$N_{v}=\left\{\left(AD\right)\left(FG\right)\left(\frac{V}{10^{12}}\right)\right\}T$$

$$N_{D}=\left(\frac{\left(AD\right)\left(FG\right)T}{A}\right)$$

The Great Pyramid of Giza contains 2.5 million cubic metres of rock [32].

## Supporting Information

S1 File Supporting Tables and Figures. S1 Table A. Comparative stone artefact density data. S1 Table B. Data from the 2011 artefact density survey. S1 Fig. A. Examples of the sample quadrates (digital photos) (photos by authors). S1 Fig. B. Photographs of diagnostic artefacts from the Messak surveys (photos by authors). S1 Fig. C. The lithic landscapes of the Messak. (photos by authors). S1 Fig. D. Landscape change and extraction of lithic raw materials (photos by authors).
(PDF)
