# Supplementary material for: Lithic Landscapes: Early Human Impact from Stone Tool Production on the Central Saharan Environment
Source: PLoS One. 2015 Mar 11;10(3):e0116482. doi: 10.1371/journal.pone.0116482 (PMC4356577; doi:10.1371/journal.pone.0116482)

Early human impact on the Central Saharan landscape through the extraction of stone for tool-making

R A Foley and Marta Mirazón Lahr

S1 Supporting information

S1 Table A. Comparative stone artefact density data.

Most archaeological surveys are focused on producing site maps, rather than systematic densities of lithics, and so although there is excellent information on the widespread distribution of lithics, there is less data which have been quantified in a way that allows for systematic comparison. The examples used here provide some indication of the range, but it should be noted that context and method differ considerably; for example Foley (1981) is a recent Holocene sample, across many sedimentary contexts, whereas Isaac (1981) is a sediment-specific survey of units known to contain artifacts.

Source	Comments	Lithic density per km^2
Foley 1981	A specifically designed artifact density survey across 600 km^2 of the Amboseli Basin, using stratified random sampling across a number of different habitats. Average densities across 8 km^2 units was 19,000 per km^2. However, this varied considerably, and one part of the sample area had an average density of 792,000 artifacts per km^2, and one sediment type had a density of 95,000 per km^2.	19000
Isaac 1981 (page 262)	A specifically designed survey across one specific set of Plio-Pleistocene sediments, know to yield artifacts and contain archaeological sites (FxJj1). Across a 14 km transect, there were small areas with high densities (c 16-60 artifacts per 25 m^2), intermediate densities (4-10 artifacts per 25 m^2), and low density (<1 artifact per 25 m^2). Data here converted to km^2 density, using 1 per 25m^2 as the	40,000

	basis for a minimum estimate.	
Olszewski et al 2010 (Figure 5)	A very extensive artifact density survey over a large area of the Nubian High Desert. 1m^2 samples taken systematically across the survey. Densities varied with distance away from the escarpment, ranging from 9 – 12 per m^2 close (< 6 kms) to the escarpment, to 0.5 – 2 per m^2 further away. This gives densities of between 12 million per km^2 and about 1 million per km^2	1,000,000 – 12,000,000

S1 Table B. Data from the 2011 artifact density survey.

Fifty 1 x 1 m quadrats were sampled, 10 each from 5 different areas. All stones larger than 15 mm (maximum dimension) were counted *in situ* and verified from digital photographs. All stones were assessed in the field for human modification, and also counted as such in the field. Samples of the digital photographs are provided below.

Square	Number stones	Number lithics
A1.1	147	91
A1.2	139	78
A1.3	177	102
A1.4	200	37
A1.5	320	66
A1.6	208	74
A1.7	179	61
A1.8	186	73
A1.9	186	60
A1.10	138	66
A2.1	253	112
A2.2	363	80
A2.3	387	96
A2.4	260	83
A2.5	219	67
A2.6	258	59
A2.7	277	46
A2.8	359	102
A2.9	493	93
A2.10	327	81
A3.1	337	123
A3.2	357	89
A3.3	340	61
A3.4	404	72
A3.5	357	57
A3.6	479	65
A3.7	482	73
A3.8	593	44
A3.9	523	97
A3.10	410	168
A4.1	481	94
A4.2	261	80
A4.3	303	92
A4.4	276	77
A4.5	273	55
A4.6	314	68
A4.7	293	76

A4.8	235	56
A4.9	339	37
A4.10	210	46
A5.1	206	64
A5.2	237	70
A5.3	336	125
A5.4	64	54
A5.5	116	53
A5.6	124	66
A5.7	115	61
A5.8	132	78
A5.9	228	79
A5.10	218	54
GENERAL SQUARE AVERAGE	282.38	75.22
GENERAL SQUARE SD	118.84	24.11

S1 Figure A Examples of the sample quadrates (digital photos)

Twelve examples of the 50 quadrats sampled. Each photograph is one metre square.

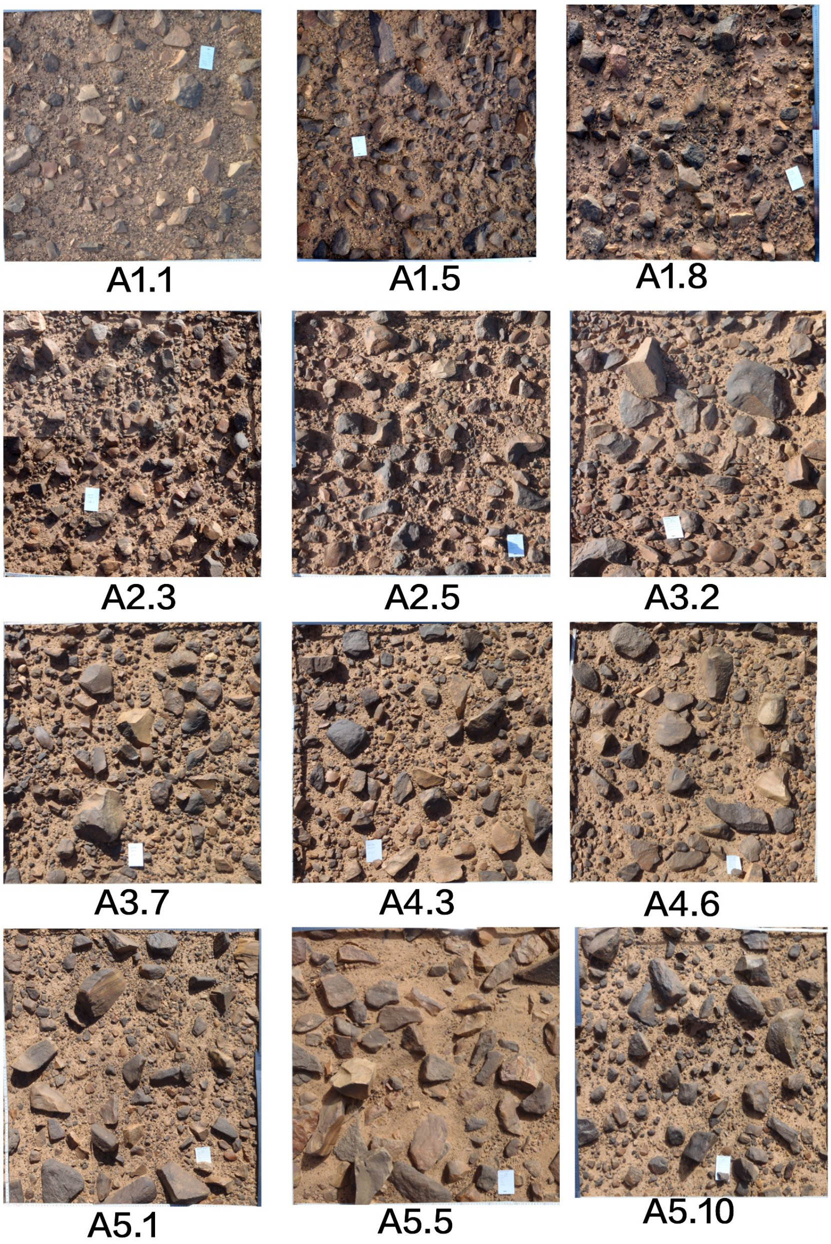

S1 Figure B Photographs of diagnostic artefacts from the Messak surveys

Detailed photographs of diagnostic stone tools from the Messak. These are typical of the Sahara MSA.

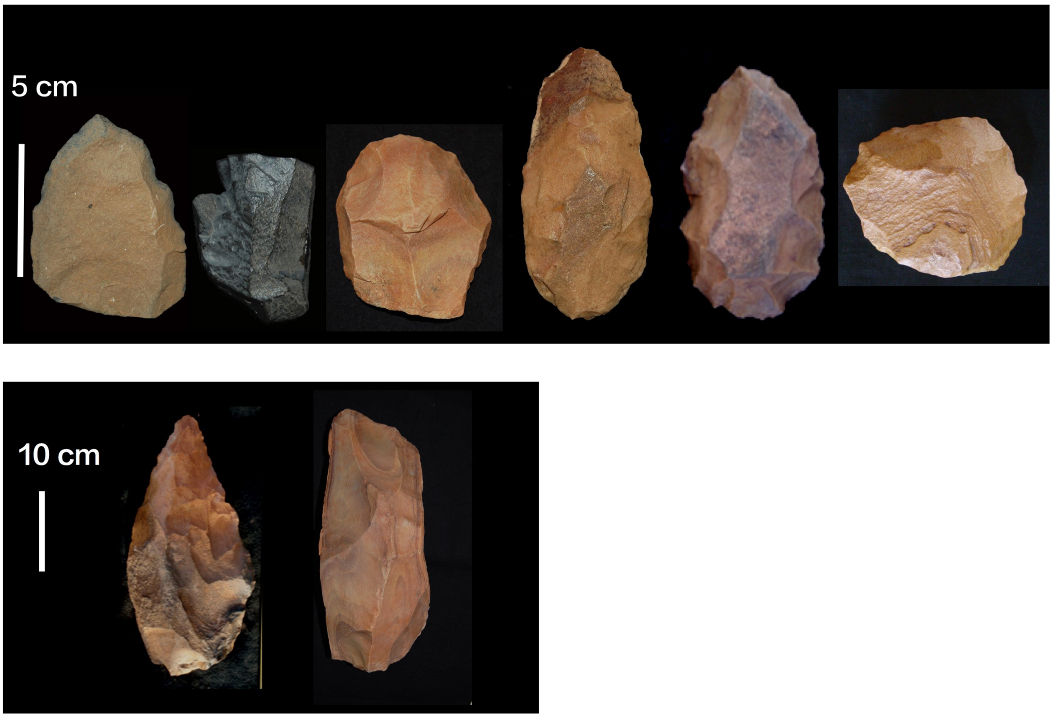

S1 Figure C The lithic landscapes of the Messak.

The photographs shows that the surface of the Messak Settafet is completely covered in stones – from gravel to large boulders, a significant portion of which consists of prehistoric stone tools.

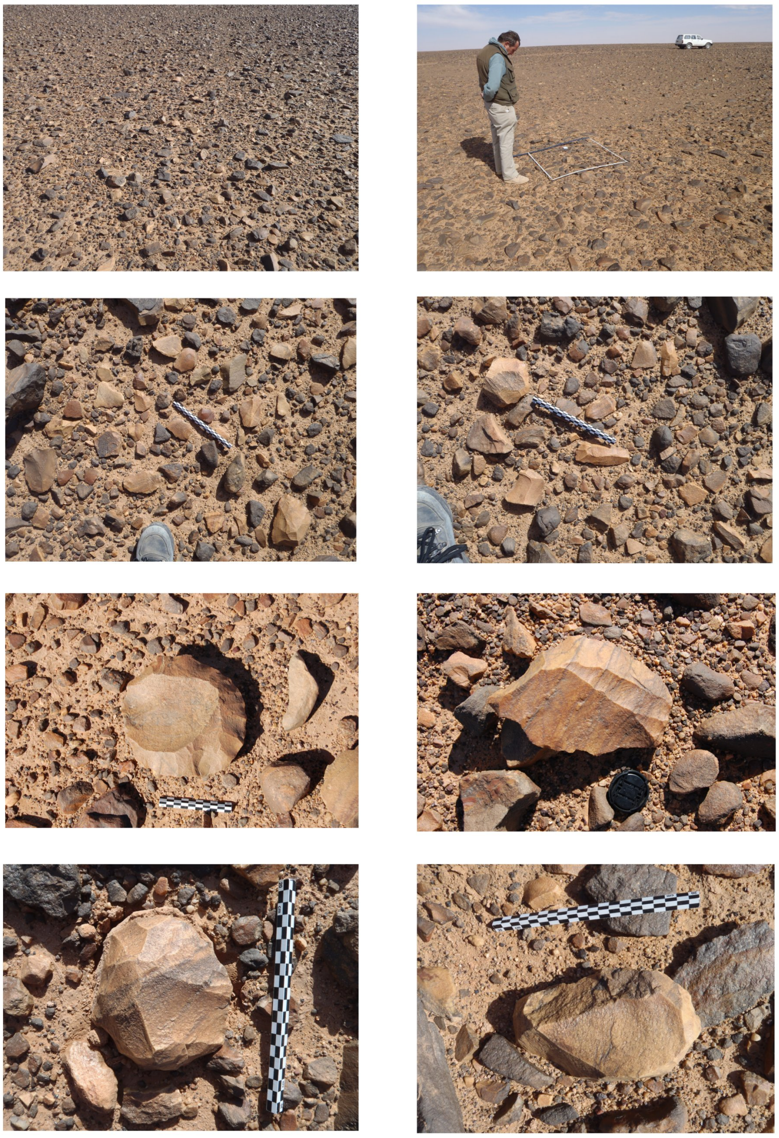

S1 Figure D. Landscape change and extraction of lithic raw materials

Examples of the effects of extraction of stones from the Messak. The top two photos show the landscape, with the small 'pits' shown as cleared areas. The middle two photos show details of examples of these. The bottom two show an area of extraction (left) and an example lithic (right).

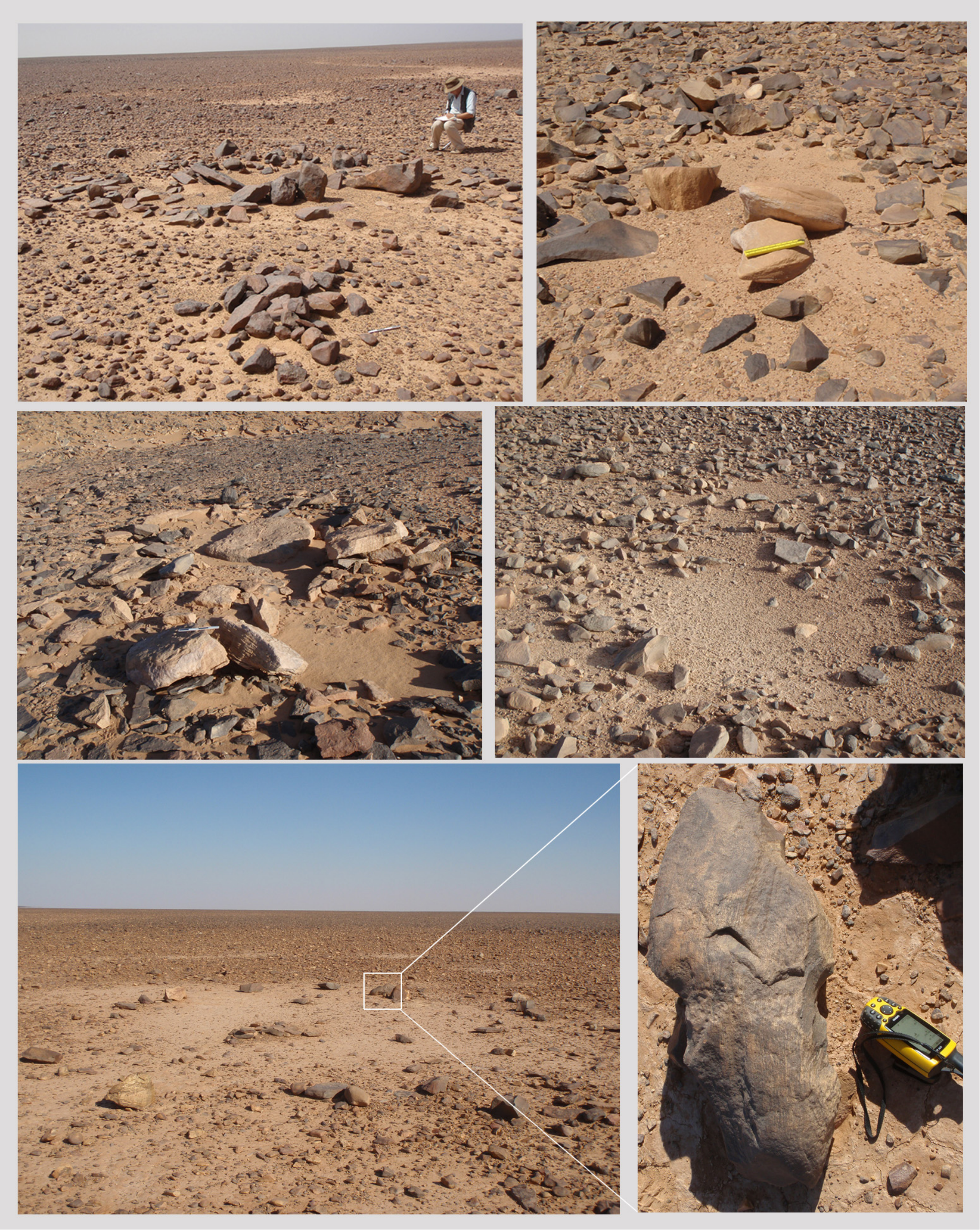

Supplement: S1 File — S1 Table A. Comparative stone artefact density data. S1 Table B. Data from the 2011 artefact density survey. S1 Fig. A. Examples of the sample quadrates (digital photos) (photos by authors). S1 Fig. B. Photographs of diagnostic artefacts from the Messak surveys (photos by authors). S1 Fig. C. The lithic landscapes of the Messak. (photos by authors). S1 Fig. D. Landscape change and extraction of lithic raw materials (photos by authors). (PDF) [file pone.0116482.s001.pdf]
